# Dietary Patterns of Treatment–Resistant Depression Patients

**DOI:** 10.3390/nu14183766

**Published:** 2022-09-13

**Authors:** Agnieszka Mechlińska, Adam Włodarczyk, Marta Gruchała-Niedoszytko, Sylwia Małgorzewicz, Wiesław Jerzy Cubała

**Affiliations:** 1 Department of Psychiatry, Faculty of Medicine, Medical University of Gdańsk, 80-952 Gdańsk, Poland; 2 Department of Clinical Nutrition, Faculty of Health Sciences with the Institute of Maritime and Tropical Medicine, Medical University of Gdańsk, 80-211 Gdańsk, Poland

**Keywords:** treatment–resistant depression, ketamine, FFQ, nutrition, diet

## Abstract

Depression is a common mental disorder that occurs all over the world with treatment resistance commonly seen in clinical practice. Ketamine exhibits an antidepressant that is more often used in the case of treatment-resistant depression (TRD) in MDD and BP. Research emphasizes that a healthy diet and the nutrients it contains can lower the risk of developing depression and form a strategy that supports conventional treatment. The aim of the study was to evaluate the patients’ diet and to analyze the effect of ketamine on food intake among patients with TRD. The study involved 15 patients suffering from treatment-resistant depression and 15 healthy volunteers. The data required for the analysis were collected using the food frequency questionnaire (FFQ) and 4-day food diaries. The study group was statistically significantly less likely to consume milk and plain milk beverages, plain white cheese, wholemeal bread, various vegetables, wine, and drinks. Our results show several disorders in the eating habits of patients with treatment–resistant depression. After the administration of ketamine, the patients consumed significantly less protein, fats, monounsaturated fatty acids (MUFA) and polyunsaturated fatty acids (PUFA), fiber, tryptophan, vitamins, and minerals compared to the control group. There is a lack of research describing the effects of ketamine on nutrition. In order to confirm the results of the study, more participants are required, and the assessment of food diaries filled in at the patient’s home with a longer interval after the last dose of ketamine as well.

## 1. Introduction

Major depression is a common mental disorder that occurs all over the world. It is estimated that 3.8% of the population suffers from depressive disorder and also that it is going to be the leading cause of the world’s burden of disease in 2030 [1]. The causes of depression are complex. They include interactions between social, psychological, and biological factors, such as changes in neurotransmitter systems, dysregulation of the hypothalamic–pituitary–adrenal axis, inflammations, and epigenetics [2,3,4,5,6]. Research shows a strong link between depression and physical health. Depression is associated with an increased risk of many chronic diseases, including cardiovascular disease, diabetes, Alzheimer’s disease, rheumatoid arthritis, and Parkinson’s disease. Therefore, it is extremely important to learn about potential preventive strategies to mitigate the adverse consequences of depression [7]. There are many pharmacological agents available that are aimed at reducing the symptoms of depression and improving the quality of life of patients. Improvement is not uncommon within a few weeks of initiating treatment with conventional antidepressants. However, in many patients struggling with depression, the expected therapeutic effects are not achieved [8,9].

Many antidepressants mainly modulate monoaminergic brain circuits (dopamine, norepinephrine, serotonin) [10]. According to CANMAT (Canadian Network for Mood and Anxiety Treatments) Clinical Guidelines for the Management of Adults with Major Depressive Disorder, first-line recommendations for pharmacotherapy for MDD are SSRIs (the selective serotonin reuptake inhibitors), SNRIs (serotonin and noradrenaline reuptake inhibitors) agomelatine, bupropion, mirtazapine, and vortioxetine. Recommended second-line medications include TCAs, quetiapine, trazodone, moclobemide, levomilnacipran, and vilazodone. MAO inhibitors and reboxetine are considered as the third-line recommendations. Due to the fact that there are many clinical features and medication characteristics that have an impact on the choice of the antidepressant, both physician expertise and an assessment of patient’s individual needs should be involved in the process of choosing the antidepressant [11]. Unfortunately, despite receiving antidepressant treatment based on evidence, up to 70% of people will still experience onerous symptoms [10].

Treatment-resistant major depression (TRD) can be defined as the lack of therapeutic response in adult patients with depressive disorders to at least two different antidepressants, which were used in the right doses for the appropriate time [12]. Among the pharmacological strategies can be distinguished: optimization; treatment with two drugs, usually with different mechanisms of action; augmentation; drug change; non-pharmacological therapies, such as electroconvulsive therapy; or attempts at other neurostimulation strategies [13,14]. Medicines with novel mechanisms of action that differ from traditional monoaminergic antidepressants offer particular hope for this group of patients. One of them is ketamine.

Ketamine is classified as an NMDA (N-methyl-D-aspartate) receptor antagonist and had been used as an anesthetic for decades before it was discovered as an antidepressant. The data from scientific studies indicate that the expression of these receptors increases in affective disorders [15]. On the other hand, it was found that the blockade of NMDAR may cause locally increased glutamate release, which results in the activation of AMPA (α-amino-3-hydroxy-5-methyl-4-isoxazolepropionic acid) receptors glycogen synthase kinase 3 phosphorylation, mammalian target of rapamycin (mTOR) signaling activation, increased production of brain-derived neurotrophic factor (BDNF), and inhibition of eukaryotic elongation factor 2 kinase [16]. Overall, by acting on this receptor ketamine induces synaptogenesis and reverses the atrophy of nerve cells in the hippocampus and prefrontal cortex [16,17]. Ketamine administration appears to be an effective treatment strategy for TRD patients. Scientific data show that among patients who formerly failed to respond to at least two antidepressants, ketamine contributed to improve their depression symptoms [18]. In addition, ketamine exhibits rapid anti-suicidal effect [11].

The therapeutic effects of ketamine on mental disorders are evident at concentrations ten times lower than the proposed concentrations needed for anesthesia [19]. Several studies have demonstrated the antidepressant efficacy of ketamine in doses of 0.5 mg/kg body weight administered intravenously as an infusion lasting 40 min, particularly in relapse prevention prophylaxis [20].

Therefore, more and more attention is paid to alternative methods of treatment that can support standard medical management and help patients more effectively. One of them is dietary treatment [21]. Findings suggest that the prevalence of depression is related to the proportion of macronutrients intake in daily life, especially the protein intake results in the reduction in the occurrence of depression [22]. Many studies show that the consumption of nutrients, such as folic acid, vitamin D, vitamin B12, omega-3 fatty acids, and zinc, may reduce the risk of mental disorders, especially depression, and improve health [23]. An important role in the pathogenesis of affective disorders is probably played by the immune and inflammatory responses and the production of free radicals. It is believed that limiting the consumption of saturated fatty acids and trans fatty acids while supplying unsaturated fatty acids and ingredients, such as antioxidant vitamins (A, C, E), zinc, and selenium, have a synergistic effect in alleviating the inflammation accompanying depression [24]. Apart from that, divalent ions (zinc, magnesium, selenium) might exert effects on the activity of cortical brain-derived neurotrophic factor, the mechanism of action of NMDA antagonists, or neuromodulation [25]. In addition, studies conducted in the group of patients with depression have shown that they have a lower average level of folate than patients not suffering from depression. Folates and B vitamins (B6, B12) are involved in the methionine metabolism cycle, which is crucial for the proper metabolism of serotonin, dopamine, noradrenaline, and phospholipids in the central nervous system. Insufficient supply of B vitamins in the diet can cause accumulation of homocysteine (hyperhomocysteinemia) and disturbances in the production of neurotransmitters (monoamines) essential for maintaining a good mood [24].

A lower-quality diet, i.e., containing processed foods; fast food; refined grains; animal fats, rich in added sugars and low in vegetables; fruit; and sources of healthy fats, such as fish, nuts, and vegetable oils, can negatively affect mental health [26].

Eating habits affect body weight. It was found that there is a positive correlation between depression and body weight. Obesity may influence depressive symptoms bidirectionally [27]. It may impact ones self-esteem per sociocultural patterns. Moreover, pro-inflammatory cytokines caused by adipocytes affect brain physiology, which may lead to occurrences of depression. Moreover, excess body weight is associated with the above-mentioned chronic diseases, which may have an impact on mental disorders. It should be noted that underweight might be related to mood disorders as well and increase the risk of depression [28]. Moreover, changes in appetite are taken into account as a symptom of depression [29]. According to the International Classification of Diseases (ICD-10) and Diagnostic and Statistical Manual of Mental Disorders (DSM-5) criteria, decreased appetite is often associated with depression [30,31]. The accompanying low mood and energy loss also cause a decreased willingness to take care of proper nutrition. Eventually it may lead to weight loss, malnutrition, and greater weakness. Antidepressant agents are the first-line treatment option for MDD, which regulates appetite and may have weight change consequences. Rapid-acting antidepressants, such as ketamine, are assumed to improve the appetite, as one of the symptoms. However, it still remains unclear how the ketamine administration influences the appetite and the body weight [32].

The main goals of the study are to assess the diet of patients suffering from treatment-resistant depression in comparison to healthy volunteers and to check how ketamine treatment affects the diet of patients with TRD.

## 2. Materials and Methods

### 2.1. Study Procedure and Participants

The study involved 15 patients with treatment-resistant depression hospitalized at the Department of Psychiatry of the University Clinical Center in Gdańsk, who were referred for ketamine treatment. The study was approved by the Independent Bioethical Committee for Scientific Research at the Medical University of Gdańsk parts of the register. Qualification for the study took place after approval by a psychiatrist. The exclusion criteria included: cancers, pregnancy, patient’s lack of cooperation, unstable health condition of the patient, and disagreement with the examination. The entire treatment process lasted ± 4 weeks and included 8 doses of the drug for each person in the study group. Ketamine was administered twice a week. The control group involved 15 volunteers who matched the study group in terms of sex and age, after the exclusion of depression. Volunteers were selected from the same province (native Polish patients and volunteers). The study was carried out during the COVID-19 pandemic (July 2021–May 2022).

### 2.2. Applied Questionaries

#### 2.2.1. Personal and Medical Data

Self-reported personal and medical information was collected from each participant (age, body weight, height, comorbidities, medications taken, etc.). The body mass index (BMI) was calculated and categorized as underweight (BMI < 18.5 kg/m^2^), normal weight (BMI 18.5–24.9 kg/m^2^), pre-obesity (BMI 25.0–29.9 kg/m^2^), obesity class I (BMI 30.0–34.9 kg/m^2^), obesity class II (35.0–39.3 kg/m^2^), obesity class III (≥40 kg/m^2^) [33].

#### 2.2.2. Exclusion of Depression

The decisive factor in qualifying for the control group was excluding depression in healthy volunteers using the 9-point Patient Health Questionnaire (PHQ-9) [34]. This questionnaire is a screening tool for the detection of depressive disorders and their severity in adults [35]. It consists of 9 questions, each of which is focused on a different symptom that may indicate depression, such as feeling sad, anhedonia, fatigue, lack of appetite, and suicidal thoughts. Each question includes 4 answers, indicating the frequency of symptoms in the last 2 weeks (“not bothering at all”–“almost every day”), scored 0–3, respectively. Twelve points is the cut-off value adopted after calculating the validation for the Polish population. Volunteers whose sum of results was <12 were qualified for the control group [34].

#### 2.2.3. Diet

To assess diet, two methods were used: The food frequency questionnaire (FFQ-6) and food dairies [36]. FFQ is validated tool for Polish population, which aims to collect information about how often 62 assortment groups of products are eaten within 12 months [37]. They represent 8 main groups of food: sweets and snacks, dairy products and eggs, grain products, fats, fruit, vegetables and grains, meat and fish products, and drinks. All listed products in the FFQ are available and often typical for Polish consumers. Participants could choose one of the following terms: Never or hardly ever”, “once a month or less often”, “several times a month”, “several times a week”, “every day”, and “several times a day” [38]. The next step was filling 4-days food diaries to evaluate more precisely what products and in what quantities were being eaten [36]. They main aim of using them was to assess how ketamine treatment affect the food intake. The diaries were entered into the “Nuvero” diet program, which made it possible to check them for macro and micronutrient content. Averaged values from 4 days were used for the analysis. The analysis focused on the following nutrients: proteins, fats, carbohydrates, fatty acids (saturated, monounsaturated, polyunsaturated, omega-3), fiber, cholesterol, sugar, tryptophan, selenium, zinc, iron, magnesium, folic acid, B12, vitamin A, vitamin C, vitamin D, vitamin E. To evaluate whether ketamine affects the diet, patients (the study group) filled in 4-day diaries twice with a minimum interval of 4 weeks (before and after ketamine treatment). Volunteers in the control group filled in the food diary once. To avoid discrepancies between the two groups due to the seasonality of products, there was an emphasis on filling the diaries in the same period.

Ultimately, food diaries from 10 patients with TRD were used for the analysis. The rest of the diaries were not made for various reasons. Ten patients submitted their diaries twice, before and after ketamine treatment (20 in total). From the control group, 11 of them were used (from volunteers appropriately matched in terms of sex and age).

#### 2.2.4. Statistical Analysis

**Figure 1:**
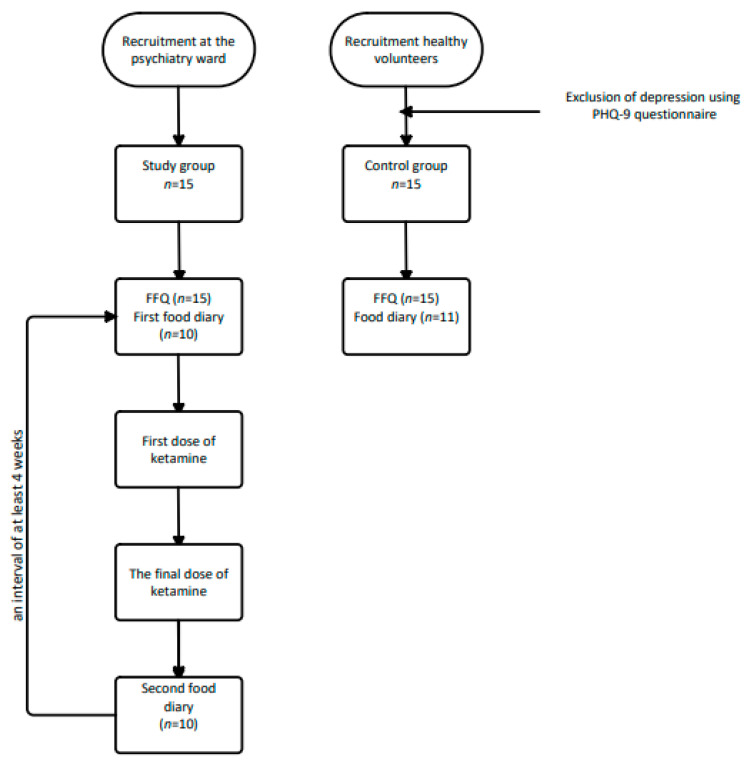
Diagram for conducting the surveys.

Statistica 13.1 Statsoft is a computer program that was used to perform the statistical analysis. The following parameters were checked: minimum and maximum values and standard deviation of BMI and age, nutrients’ arithmetic mean of 4 days, and statistical significance (*p*-value, *p* < 0.05). The *p*-value for the food diaries was determined using the Student’s t-test, for the FFQ questionnaire using the Mann–Whitney U Test. Figure 1 shows a diagram for conducting the surveys.

## 3. Results

### 3.1. Participants’ Characteristics

There were 15 participants in both groups (8 women and 7 men). Their main characteristics are shown in Table 1. A total of 40% of participants in the study group and 46.7% in the control group were overweight. The percentage of people with obesity was higher in the study group (26.7% vs. 6.7%). Among patients with depression disorder, there were no cases of being underweight.

### 3.2. Food Frequency Questionnaires Results

The order in which the results are presented corresponds to the order of the food products presented in FFQ.

#### 3.2.1. Consumption of Sweets and Snacks

There were no statistically significant differences in the frequency of consumption of sweets and snacks between the study group and the control group. A total of 40% of depression disorder patients declared that they consumed sugar “never or hardly ever”. The same frequency of sugar consumption declared 46% of healthy volunteers without depression (Table 2).

#### 3.2.2. Consumption of Dairy Products and Eggs

**Figure 2:**
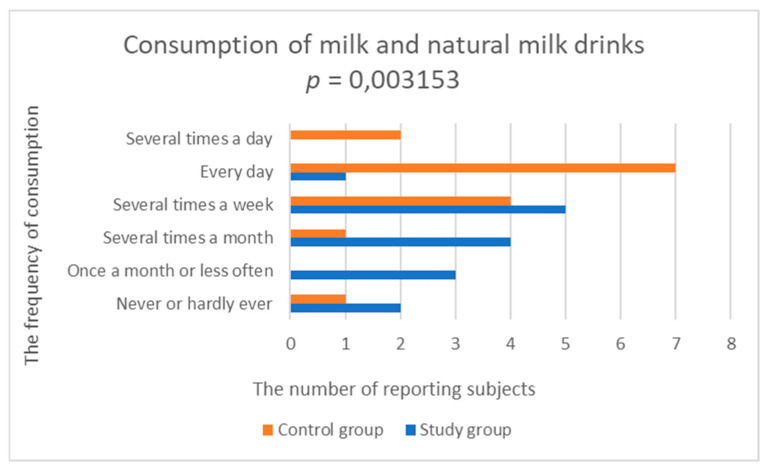
Comparison of milk and natural milk drinks consumption in the study and control groups.

**Figure 3:**
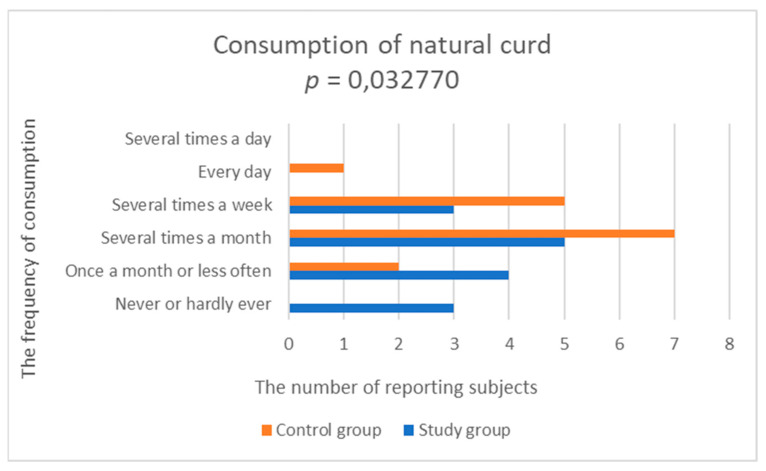
Comparison of natural curd consumption in the study and control groups.

In 87% of the study group, dairy and eggs were not included in the daily menu. In the control group, 60% of respondents declared consuming milk and natural milk drinks every day or even several times a day. A statistically significantly lower frequency of consumption of milk and natural milk drinks was demonstrated in the study group compared to the control group (study group; 3.0 ± 1.195 vs. control group; 4.429 ± 1.284, *p* = 0.003153) (Figure 2, Table 2). Consumption of natural curd was significantly lower in the study group, compared to the control group (study group; 2.533 ± 1.356 vs. control group; 3.429 ± 0.756, *p* = 0.032770) (Figure 3, Table 2).

#### 3.2.3. Consumption of Grain Products

**Figure 4:**
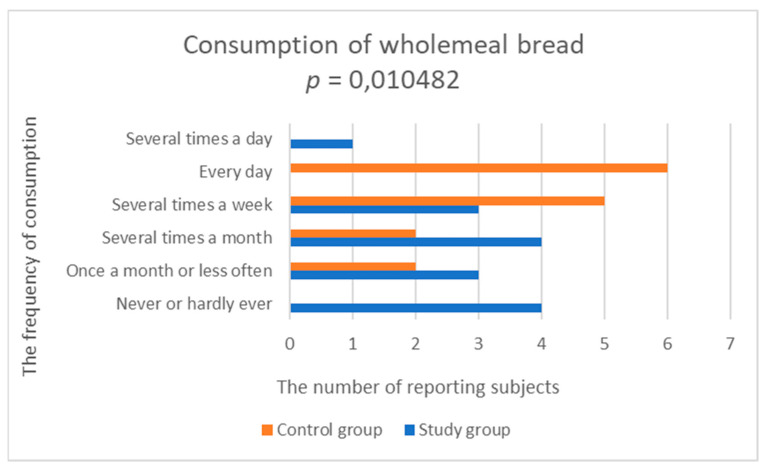
Comparison of wholemeal bread consumption in the study and control groups.

A product that was consumed more often by patients with TRD was white bread. A statistically significantly lower frequency of consumption of wholemeal bread was found in the study group, compared to the control group (study group; 2.667 ± 1.447 vs. control group; 4.0 ± 1.109, *p* = 0.010482) (Figure 4, Table 2).

#### 3.2.4. Consumption of Fats

In both groups, the most consumed foods were oils and butter. There was no statistically significant difference in the frequency of consumption of fats between the two groups (Table 2).

#### 3.2.5. Consumption of Fruit

40% of the study group ate fruit mostly several times a week. Only 20% of patients declared fruit consumption each day. In the control group, 40% of people declared fruit consumption every day. No statistically significant differences were found between groups (Table 2).

#### 3.2.6. Consumption of Vegetables and Grains

**Figure 5:**
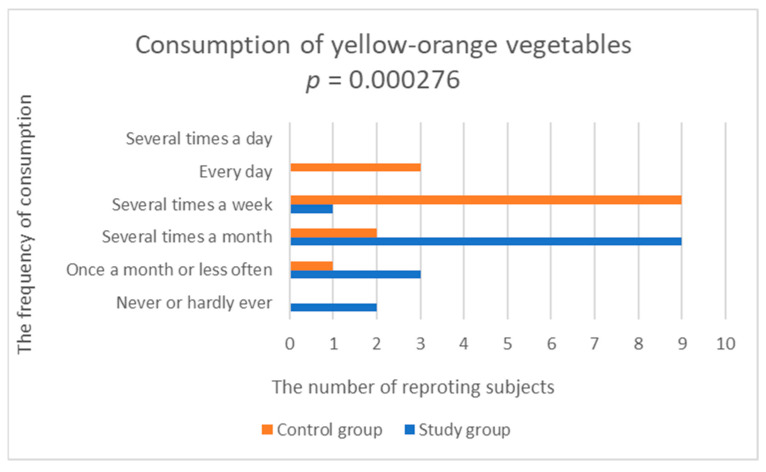
Comparison of yellow-orange vegetables consumption in the study and control groups.

**Figure 6:**
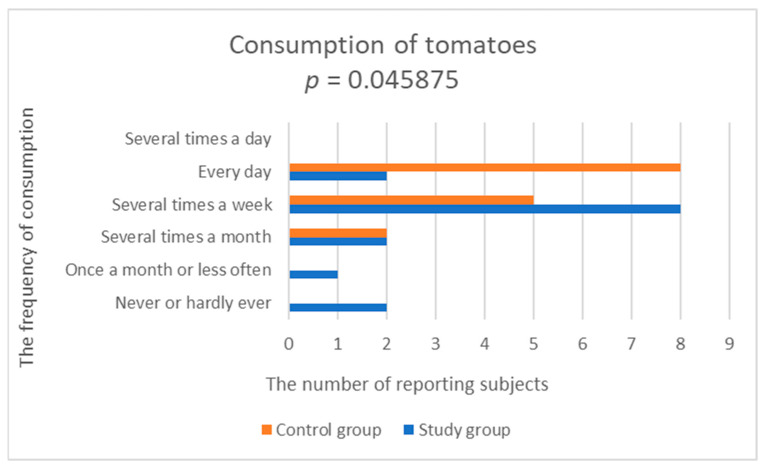
Comparison of tomatoes consumption in the study and control groups.

**Figure 7:**
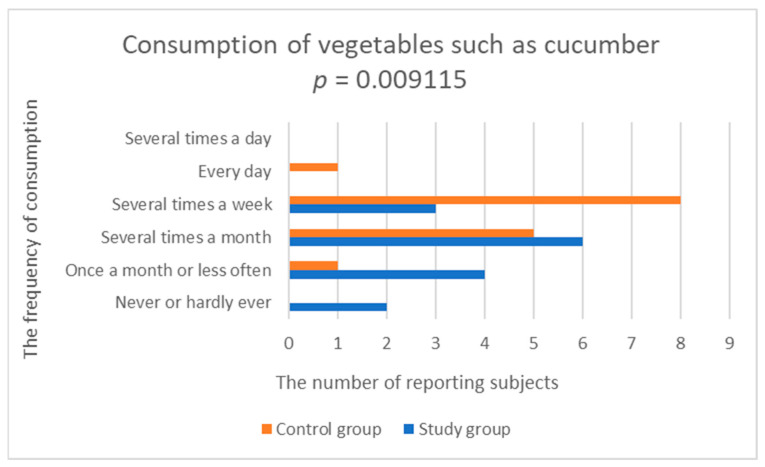
Comparison of vegetables, such as cucumber consumption in the study and control groups.

33% of patients with depression declared eating vegetables several times a week, and the same number of patients said that they ate vegetables every day. Only 13% of patients ate vegetables several times a day. In the control group, 47% of people ate vegetables every day and 27% ate vegetables several times a day. The study group was found to have statistically significantly lower frequency of consumption of yellow-orange vegetables (study group; 2.533 ± 0.915 vs. control group; 3.929 ± 0.829, *p* = 0.000276), tomatoes (study group; 3.4 ± 1.352 vs. control group; 4.357 ± 0.745, *p* = 0.045875) and vegetables, such as cucumber (fresh cucumbers, squash, zucchini, pumpkin, eggplant) (test group; 2.6 ± 1.056 vs. control group; 3.643 ± 0.745, *p* = 0.009115), compared to the control group (Figure 5, Figure 6 and Figure 7, Table 2).

#### 3.2.7. Consumption of Meat and Fish Products

There were no significant differences in the consumption of these products.

#### 3.2.8. Consumption of Beverages

**Figure 8:**
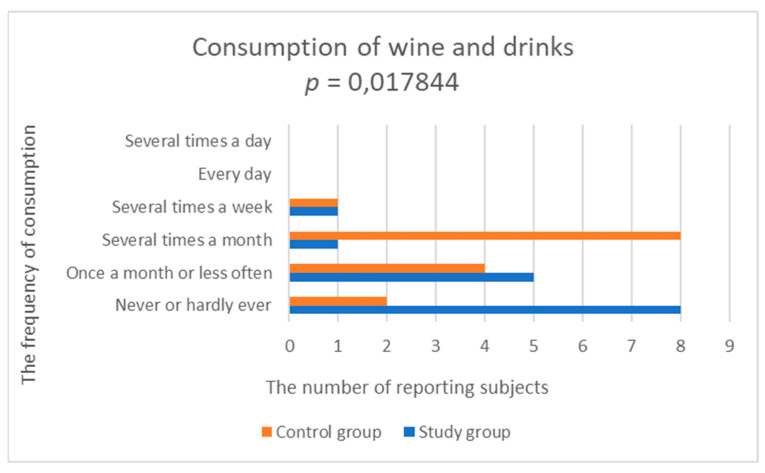
Comparison of wine and alcohol drinks consumption in the study and control groups.

Beverages did not appear in the daily diet of TRD patients. Alcoholic beverages were the least frequently consumed. In the control group, alcoholic beverages were more common. A statistically significantly lower frequency of consumption of wine and alcohol drinks was demonstrated in the study group, compared to the control group (study group; 1.667 ± 0.9 vs. control group; 2.5 ± 0.855, *p* = 0.017844) (Figure 8, Table 2).

### 3.3. Statistical Analysis of Food Diaries. The Ketamine Treatment Correlated with Nutrients

#### 3.3.1. Energy Consumption Analysis

In the study group before ketamine treatment, the mean amount of energy consumed was 1848.34 kcal ± 885.49, and after treatment, these values did not differ statistically. There was no statistically significant difference in energy consumption between the group after administration of ketamine (1629.68 kcal/day ± 829.14) and the control group (2216.08 kcal/day ± 596.84, *p* > 0.05) (Table 3).

#### 3.3.2. Analysis of Macronutrient Intake: Proteins, Fats, and Carbohydrates

**Figure 9:**
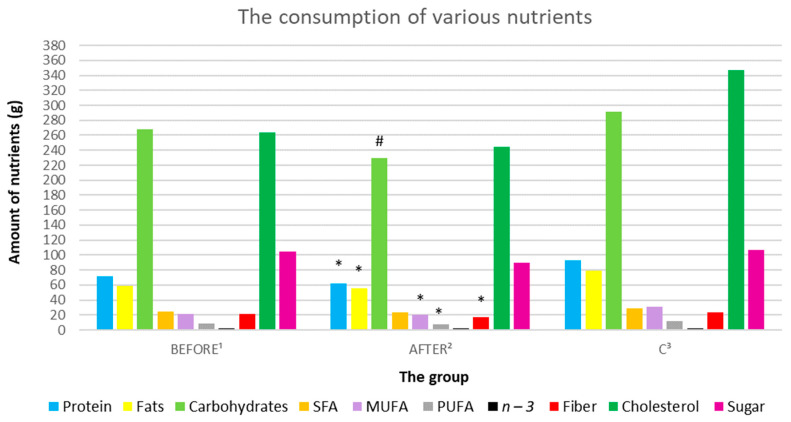
Average daily consumption of various nutrients by groups. ^1^ study group prior to ketamine administration; ^2^ study group after administration of ketamine; ^3^ control group; # represents a statistically significant result *p* < 0.05 for the group of patients after administration of ketamine, compared to the group before administration; * indicates a statistically significant result *p* < 0.05 for the group of patients after administration of ketamine, compared to the control group.

The study group showed significantly lower carbohydrates consumption after ketamine treatment, compared to the group before treatment (sequentially; 268.18 g/day ± 122.92 vs. 229.68 g/day ± 142.76, *p* = 0.037566). The consumption of protein and fats in the study group before and after ketamine did not differ statistically significantly (Figure 9, Table 3).

A statistically significant lower consumption of both protein and fats was demonstrated in the study group after administration of ketamine, compared to the control group (in sequence; 62.23 g/day ± 24.83 vs. 93.34 g/day ± 20.53, *p* = 0. 005365; 55.06 g/day ± 26.43 vs. 79.13 g/day ± 25.5, *p* = 0.04707). There was no statistically significant difference in carbohydrate consumption in the study group after administration of ketamine, compared to the control group (Figure 9, Table 3).

#### 3.3.3. Analysis of Fatty Acid Consumption

In the study group before the administration of ketamine, no statistically significant differences in the consumption of fatty acids (saturated fatty acids (SFA), omega-3) were found in comparison to the control group and after ketamine treatment. Patients after administration of ketamine showed statistically significantly lower consumption of MUFA and PUFA acids, compared to the control group (sequentially; 20.19 g/day ± 9.56 vs. 31.28 g/day ± 10.95, *p* = 0.0236; 7.29 g/day ± 3.31 vs. 12.13 g/day ± 4.21, *p* = 0.009078) (Figure 9, Table 3).

#### 3.3.4. Analysis of Dietary Fiber Intake

In the study group before the administration of ketamine, no statistically significant differences were found in the consumption of fiber, compared to the control group and the ketamine-treated group. Patients after administration of ketamine showed a statistically significantly lower consumption of fiber, compared to the control group (sequentially; 16.58 g/day ± 6.92 vs. 23.79 g/day ± 4.71, *p* = 0.01101) (Figure 9, Table 3).

#### 3.3.5. Analysis of Cholesterol and Sugar Consumption

Patients in the study group did not differ significantly in cholesterol and sugar consumption, either before and after ketamine treatment. There were no significant differences in the consumption of cholesterol and sugar between the study group and the control group (Figure 9, Table 3).

#### 3.3.6. Tryptophan Intake Analysis

**Figure 10:**
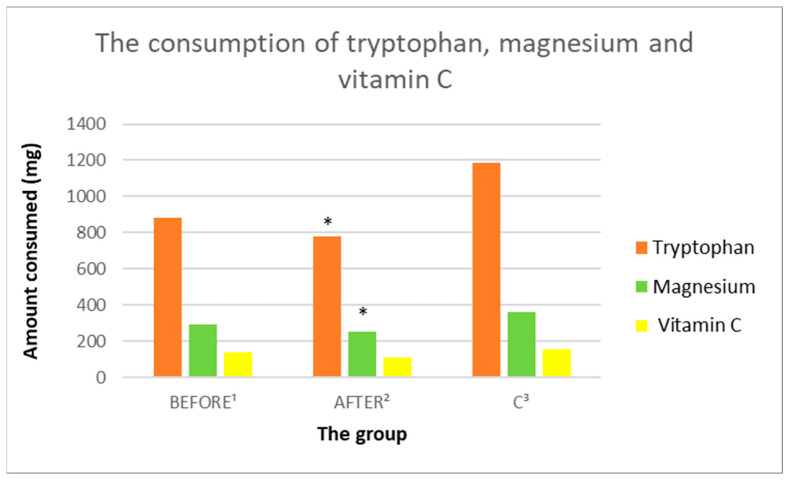
Average daily intake of tryptophan, magnesium, and vitamin C by groups. ^1^ study group prior to ketamine administration; ^2^ study group after administration of ketamine; ^3^ control group; * indicates a statistically significant result *p* < 0.05 in the group after administration of ketamine, compared to the control group.

In the study group before the administration of ketamine, no statistically significant differences were found in the consumption of tryptophan, compared to the control group and after treatment with ketamine. Patients after administration of ketamine showed statistically significantly lower intake of tryptophan, compared to the control group (consecutively; 776.2 mg/day ± 306.24 vs. 1185.53 mg/day ± 297.63, *p* = 0.005833) (Figure 10. Table 3).

#### 3.3.7. Analysis of Vitamin B9 and B12 Intake

**Figure 11:**
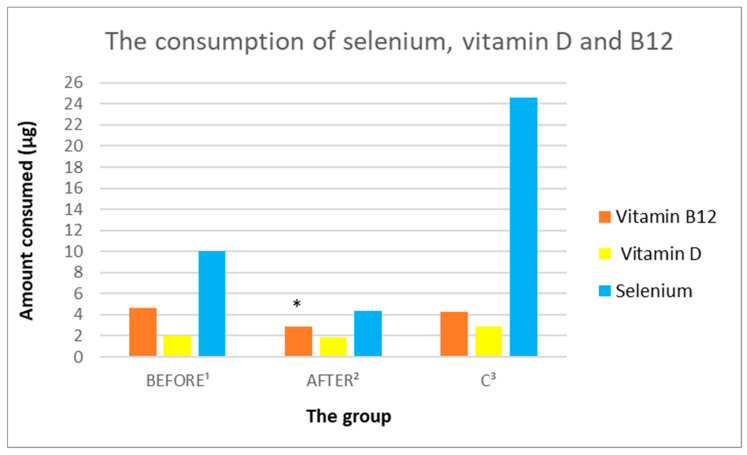
Average daily intake of vitamin D, B12, and selenium by groups. ^1^ study group prior to ketamine administration; ^2^ study group after administration of ketamine; ^3^ control group; * indicates a statistically significant result *p* < 0.05 in the group after administration of ketamine, compared to the control group.

**Figure 12:**
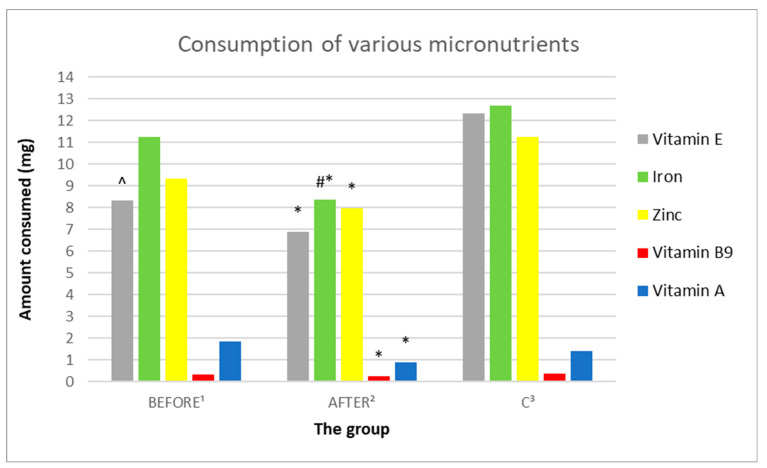
Average daily zinc, iron, vitamin E, B9, and A intake by groups. ^1^ study group prior to ketamine administration; ^2^ study group after administration of ketamine; ^3^ control group; ^ means a statistically significant result *p* < 0.05 for the pre-ketamine group, compared to the control group; # represents a statistically significant result *p* < 0.05 for the group of patients after administration of ketamine compared to the group before administration; * indicates a statistically significant result *p* < 0.05 for the ketamine group, compared to the control group.

In the study group before the administration of ketamine, no statistically significant differences were found in the intake of vitamins B9 and B12, compared to the control group and after ketamine treatment (Figure 11 and Figure 12, Table 3). Patients after administration of ketamine demonstrated statistically significantly lower intake of vitamins B9 and B12, compared to the control group (sequentially; 255.92 µg/day ± 126.25 vs. 382.97 µg/day ± 126.77, *p* = 0.033071; 2.88 µg/day ± 1.8 vs. 4.3 µg/day ± 1.27, *p* = 0.049312).

#### 3.3.8. Analysis of the Consumption of Vitamins C, A, D, E

In the study group before the administration of ketamine, no statistically significant differences in the consumption of vitamins C, A, and D were found, compared to the group after ketamine treatment (Figure 10, Figure 11 and Figure 12, Table 3). The study group demonstrated statistically significantly lower intake of vitamin E both before and after ketamine treatment, compared to the control group (sequentially; 8.31 mg/day ± 3.95 vs. 12.33 mg/day ± 2.56, *p* = 0.011688; 6.9 mg/day ± 3.21 vs. 12.33 mg/day ± 2.56, *p* = 0.000378). A statistically significantly lower intake of vitamin A was demonstrated in the study group after ketamine treatment, compared to the control group (respectively; 904.84 µg/day ± 329.47 vs. 1416.43 µg/day ± 504.93, *p* = 0.013656).

#### 3.3.9. Analysis of the Consumption of Micronutrients; Selenium, Zinc, Magnesium, Iron

In the study group before ketamine treatment, no statistically significant differences in the consumption of selenium, zinc, and magnesium were found, compared to the group after treatment with ketamine. The only micronutrient consumed in a statistically significantly higher concentration in the study group before treatment, compared to after treatment with ketamine was iron (sequentially; 11.26 mg/day ± 5.91 vs. 8.35 mg/day ± 4.24, *p* = 0.029401). Patients after administration of ketamine showed statistically significantly lower intake of iron, magnesium and zinc, compared to the control group (sequentially: 8.35 mg/day ± 4.24 vs. 12.68 mg/day ± 2.35, *p* = 0.008538; 253.84 mg/day ± 120.38 vs. 362.29 mg/day ± 54.33, *p* = 0.014033; 7.98 mg/day ± 3.81 vs. 11.23 mg/day ± 1.81, *p* = 0.020072) (Figure 10, Figure 11 and Figure 12, Table 3).

#### 3.3.10. Assessment of Coverage of the Daily Requirements for Certain Nutrients

None of the people participating in the study covered the required daily intake of vitamin D and selenium. The only nutrient that required intake covered by 100% of the volunteers in the control group were vitamin A, zinc, and tryptophan. In the group after administration of ketamine, the coverage of the daily requirement for most nutrients decreased (Table 4).

## 4. Discussion

In the study group, we observed a lower consumption of products, such as wholemeal bread, milk, natural milk drinks, and natural curd. Moreover, the diversity and frequency of consumed vegetables containing many bioactive anti-inflammatory and neuroprotective compounds among patients with depression were significantly lower [39].

In our study, the analysis of food diaries showed a tendency toward lower intakes of essential macronutrients (proteins, fats, and carbohydrates) in patients with depressive disorder, compared to healthy volunteers. After treatment with ketamine, statistically significant lower values of the mean consumption of protein and fat were recorded, compared to the control group, and the difference in carbohydrate consumption in this group was statistically lower, compared to the group before the administration of ketamine.

There was no statistically significant difference in the frequency of consumption of MUFA and PUFA sources, but we observed a lower frequency of the consumption of olives, grains, or avocados in the group of TRD patients. The consumption of omega-3 fatty acids performed a tendency to increase in the control group, compared to patients with depression. After treatment with ketamine, the patients consumed statistically significantly less total fat; unfortunately, they did not show statistically significant differences in the consumption of SFA, compared to the control group.

Even though in both groups coverage of the daily fiber requirement (25 g assumed as the lower limit) was very low (less than 50% of the groups covered the daily requirement for fiber), its share in the diet was greater in the control group [40].

The consumption of tryptophan showed an increasing tendency in the group of healthy volunteers, compared to patients with depression [41]. An interesting observation was the significantly lower consumption of tryptophan in the group of patients with depression after treatment with ketamine, compared to healthy people.

The results showed a significant reduction in vitamins in the patients’ diet after the treatment with ketamine. Most healthy participants consumed a sufficient amount of vitamin E and 100% of the group met the standards for vitamin A daily intake. Nobody met the requirement for vitamin D taking into account its supply with the diet. Half of the control group was supplemented with vitamin D. In the study group it was 27%. No statistically significant differences in the consumption of vitamins B12 and B9 were found in the group of patients with depression and healthy participants, but 91% of healthy participants covered the daily requirement for vitamin B12 and only 70% of patients with TRD. After the administration of ketamine, the consumption of both vitamins was lower than in healthy subjects. In addition, the coverage of the daily requirement for these vitamins decreased by about 10% after the treatment.

The consumption of zinc and magnesium tended to be higher among healthy subjects. Coverage of both zinc and magnesium requirements also differed. In the control group, 100% of participants covered the daily requirement for zinc and 73% for magnesium. In the study group, before ketamine, it was 70% and 40%, respectively. Nobody in the group of patients with TRD, both before and after ketamine, covered the requirement for selenium. In the control group, the average amount of selenium consumed with the diet was almost six times higher than in the group after ketamine treatment. Furthermore, the consumption of iron was statistically significantly lower in the group of patients after treatment with ketamine, compared to the group before treatment and in the control group. Less than half of the people in each group covered the daily requirement for this nutrient.

The study has several strengths. It is a unique study to evaluate the effects of ketamine on nutrient intake. Two recommended methods of assessing the consumption of products were used: the FFQ questionnaire and 4-day dietary diaries, which allowed for comparisons and analysis between participants [36]. The control group was matched in terms of gender and age, and the collection of data from this group took place as close as possible to the study group to avoid differences resulting from the seasonality of food products. In addition, the control group required qualifications based on the PHQ-9 questionnaire, a validated tool, which ensured the exclusion of people with symptoms indicative of depressive disorders [34]. The study also has some limitations. The first and most basic is the small number of people in the study group. The group was strictly defined, so fewer patients could be qualified for the study. This influenced the number of assessed FFQ questionnaires and food diaries, which for various reasons were not returned in some cases. Another limitation is that nutrient intake assessments were made shortly after ketamine treatment was completed. This was due to the patients being on the ward, as there was too much risk of not completing the study after the patient had left the hospital. There are no publications that investigate the effect of ketamine on diet over a long follow-up since the last dose of the drug. Furthermore, while being on the ward, the patients consumed hospital meals. It was not their natural home environment—in this case, the products, meals, and balance could be different. All data were collected during the COVID-19 pandemic. Thus, the results obtained refer to a pandemic dietary pattern that may vary from a regular one. When it comes to food diaries, there is a risk of underestimating or misrepresenting the amounts consumed in both groups, which could have influenced the results. Additionally, the data necessary to calculate BMI (height and weight) was taken from the participants’ self-answers.

Eating habits can play an important preventive role and support conventional treatment of depression [42]. In a study by Lassale et al. [43], it was revealed that eating patterns, such as the Mediterranean diet, DASH, and vegetarian diets, have a positive effect on depressive disorders through anti-inflammatory and cardiovascular support. We observed that after the ketamine treatment, the consumption of essential nutrients decreased. A possible interpretation of these results is the supposition that ketamine suppresses the appetite or intensifies the before-treatment weakness. Lower intake of the main nutrients induced by treatment affects the quality and balance of the requirement for both macro and micronutrients, which seems to be able to influence the course of depression [44]. Lack of appetite or overeating are some of the depression symptoms [45]. Patients suffering from depression often experience changes in the perception of appetite, which may also be related to the medications they take [46]. This may explain why the control group consumed statistically non-significantly more energy (kcal) and nutrients, compared to patients with TRD.

A high consumption of vegetables, fruit, whole grains, and fish is associated with a reduced risk of depression [39]. The research show that patients consumed fewer nutritional products than healthy subjects. Patients treated with ketamine consumed the least fiber, which may cause dysbiosis and a negative impact on the effectiveness of the therapy [47]. Less frequent consumption of wholemeal bread could influence the intake of fiber, minerals, and group B vitamins [48]. Overall, more attention is paid to the beneficial influence of fiber on immune and anti-inflammatory functions, exerted by influencing the gut microbiome and the gut–brain axis [25,49,50]. Some bacteria are able to produce neuroendocrine hormones or neuroactive compounds, which are involved in crucial aspects of neurotransmission. GABA (gamma-amino butyric acid) the major inhibitory neurotransmitter of mammalian central nervous system can be synthetized by some species of Lactobacilli and Bifidobacteria [51]. There is strong evidence that changes in GABA neurotransmission can increase the risk of developing depression. Furthermore, it is said that higher intake of dietary fiber lowers the risk of cardiovascular disease and type 2 diabetes mellitus; modulates glycemic control; and regulates gut health, appetite, and body weight. All these factors can contribute to the development or worsening of symptoms of depression [52].

It has been hypothesized that the overconsumption of sugar can cause brain adaptations involving many different neural systems, consecutive changes in behaviors, and finally increase the risk of depression via several plausible biological pathways, for instance, a higher hypothalamic–pituitary–adrenal (HPA) axis reactivity leading to dysregulation of the stress response, and obesity induced heightened low-grade inflammation, etc. [53,54]. The analysis of food diaries did not show any statistically significant differences in the consumption of simple sugars in the studied groups; both groups consumed sweet snacks and drinks willingly. Knüppel et al. [55] reported that sugar intake from sweet food/beverages increases the chance of incident mood disorders in men. Their findings indicate that policies promoting the reduction in sugar intake could further support primary and secondary prevention of depression.

Food products that are a source of fats were not significantly different consumed by the study group, but it could be noted that the frequency of consumption of olives, grains, or avocados was lower in the group of TRD patients. Due to the anti-inflammatory effects in the diet monounsaturated and polyunsaturated fatty acids are beneficial nutrients. Especially omega-3 acids have a proven positive role on the nervous system [40,41]. Taking into account the health benefits of omega-3 on the nervous system with low consumption of oily fish as their main source, supplementation with these acids in all groups remains to be considered. This is, however, considered to be especially advantageous in patients treated with antidepressants. On the other hand, saturated fatty acids have a strong systemic pro-inflammatory effect in the body, and thus might have a negative impact on the neurobiological pathways leading to depression and the aggravation of the symptoms [56]. After treatment with ketamine, there were no differences in the consumption of SFA, compared to the control group, which may have an antagonistic effect on the effectiveness of the depression treatment. The sources of fats are dairy products as well. Patients with TRD consumed less dairy than healthy volunteers. Recent scientific evidence indicates that although milk fat is naturally rich in SFA, it is simultaneously a source of natural bioactive components with beneficial effects on human health, for example butyric acid helps maintain health of gut microbiota [57]. Other milk fats can modulate inflammation and show beneficial effects on inflammatory chronic diseases, which are able to cause and worsen symptoms of depression. It also seems to be advantageous to add dairy products to TRD patients’ diet because they are a good source of easily digestible protein, especially Whey protein. Whey protein-derived peptides have antioxidant properties, they inhibit the production of proinflammatory cytokines. In addition, dairy products provide crucial micronutrients (calcium, phosphorus, selenium, magnesium, and zinc) and vitamins (A, D) [58]. In our study, less consumption of this kind of products among the patients’ group may give some explanation why the above-mentioned minerals and vitamins achieved worse levels in daily diet, in comparison to the control group. It should be noted that the most effective way to deliver vitamin D is through skin synthesis. In the Polish climate, this production during the autumn–winter–spring months is insufficient. Therefore, its supplementation is important here. The results indicate a significant risk of deficiencies in both groups, especially in the study group, which may increase the risk of developing depression and aggravate its course and weaken the response to treatment [59].

In addition, healthy participants more often consumed dairy products (milk, natural curd), and wholemeal bread provided tryptophan (a source of a serotonin precursor and “protect” against the development or “improve” the course of depression) [41]. The tryptophan can be found also in eggs, meat, fish, poultry, nuts, and seeds. With regard to Lindseth et al. [60], higher doses of dietary tryptophan resulted in significantly less depression and better mood states. Furthermore, eating less dietary tryptophan caused more irritability and anxiety in comparison to the same individuals when they consumed more tryptophan. The ketamine treatment resulted in a decreasing amount of dietary tryptophan, not significantly in comparison to the before-ketamine-treated group, but it should be taken into account that it may lead to possible deficiencies.

Deficiencies of this vitamin are associated with psychiatric disorders [47]. After treatment with ketamine, the intake of vitamins A and E was significantly lower than in healthy volunteers. The study group showed a higher average intake of vitamin A after the diaries analysis, although only 60% of them provided it in accordance with adequate daily intake, which may be a factor influencing the development of depression symptoms. Moreover, the coverage of the daily vitamin B9 requirements in both groups is very low (maximum 40% of the study population) and poses a risk of deficiencies [61]. This can be explained by a reduction in the amount of food and products rich in these vitamins, as a result of treatment with ketamine. Patients with TRD consumed less amounts of vegetables, especially tomatoes, yellow-orange colored fruit, and others, such as cucumber, which are good sources of these nutrients. It should be noted that these products are easily available food items in Poland. According to the Household Budget Survey carried out by Central Statistical Office in 2020, these vegetables are often bought by consumers [62]. Inadequate coverage of the daily requirement for vitamins after ketamine treatment may be important diet-dependent factors affecting the risk of TRD and the severity of its symptoms.

It seems necessary to control the consumption of B vitamins among patients with depression, especially during treatment [61,63]. On the other hand, Markun et al. [64] did not confirm in their review that supplementation of vitamin B12 is effective for cognitive function and depressive symptoms. Participants did not show differences in the frequency of consumption of meat products, although the control group significantly more frequently consumed dairy products that were a source of cobalamin. With regard to B9 vitamin, the higher consumption of vegetables could contribute to better levels of folic acid among healthy subjects. Due to the fact they take an important part in homocysteine metabolism, which is said to be associated with depression, it seems that taking care of their proper consumption with the diet or possible supplementation could be helpful for TRD patients.

There is a significant risk of the consequences of magnesium, zinc, and also selenium deficiencies. Styczeń et al. [65] confirmed in their study the correlation between depression and zinc deficiency. Supplementation with this element reduced the severity of symptoms and improved the response to treatment, especially in patients with treatment-resistant depression [65]. Similar conclusions were drawn for magnesium by Tarleton et al. [66]; low levels were associated with the severity of symptoms. According to Issn’s and Chad’s results, selenium intake is most strongly related to depression among all the dietary factors considered [67]. It has been shown that iron deficiency may negatively affect the transmission of signals by neuro-transmitters in the central nervous system, leading to mental health disorders in the form of depressed mood and an increased risk of depression [68]. Moreover, since the consumption of iron was not sufficient, there is a risk of the appearance of symptoms additionally caused by anemia, as well as the intensification of depressive symptoms. According to Velázquez-Alva et al. [46], there is a strong association between poor nutritional status and depression. Lower consumption of macronutrients, minerals, vitamins, and energy in many patients and not meeting the daily requirements, which increase after treatment with ketamine, pose a significant risk of developing malnutrition among patients with TRD, which may have a more negative impact on depression symptoms and achieving remission of the disease.

Scientific literature focuses increasingly on the impact nutrition has on the risk of developing depressive disorders. The role of vitamins, minerals, and macronutrients is emphasized during the disease [69,70]. Proper eating habits can be a non-pharmacological alternative supporting patients in achieving remission, which may be of significant importance for patients with treatment-resistant depression. However, there are no reports on the effect of ketamine treatment on eating behavior. Future pieces of research should involve a greater number of participants. The assessment of food diaries filled in at the patient’s home with a longer interval after the last dose of ketamine would be required.

## 5. Conclusions

Our results show several disorders in the eating habits of patients with treatment-resistant depression. Additionally, an increase in the deficiencies in the patient’s diet as a result of ketamine treatment was observed. Numerous deficiencies modulating nutrients, both the immune and nervous systems, may have a significant impact on the risk of developing TRD and the effectiveness of its treatment. Therefore, one of the elements of multidisciplinary care for patients with treatment-resistant depression should be a dietitian.

## Figures and Tables

**Table 1:** Characteristics of groups.

	S *n* = 15	C *n* = 15
AGE (SD)	35.6 (11.5)	35.6 (11.6)
SEX (Woman)	8 (53.3%)	8 (53.3%)
SEX (Man)	7 (46.6%)	7 (46.6%)
BMI (SD)	27.8 (5.8)	25.3 (4.4)

S—the study group; C—the control group; SD—standard deviation.

**Table 2:** The average frequency of consumption of product groups in the study and control groups.

	S ^1^ (SD)	C ^1^ (SD)	*p*-Value ^2^
Sugar	3.067 (2.017)	2.857 (1.956)	0.846694
Honey	2.400 (1.682)	1.929 (1.072)	0.813179
Chocolates and chocolate products	3.667 (0.900)	3.786 (0.975)	0.714830
Non-chocolate candies	3.067 (1.624)	2.714 (1.139)	0.620867
Biscuits	2.533 (1.356)	3.071 (0.730)	0.093216
Ice cream and pudding	2.067 (0.961)	2.571 (0.852)	0.171789
Salty snacks	2.667 (1.113)	2.214 (0.802)	0.251712
Milk and natural milk drinks	3.000 (1.195)	4.429 (1.284)	0.003153
Sweetened milk drinks	2.733 (1.033)	2.714 (1.069)	0.982865
Natural curd	2.533 (1.060)	3.429 (0.756)	0.032770
Flavored curds	2.067 (1.100)	2.071 (1.141)	1.000000
Cheeses	3.333 (0.724)	4.071 (0.997)	0.076919
Eggs	3.000 (1.000)	3.571 (0.756)	0.171789
Wholemeal bread	2.667 (1.447)	4.000 (1.109)	0.010482
White bread	3.533 (1.407)	3.357 (1.277)	0.747192
Coarse-grained groats	2.400 (0.986)	2.857 (0.949)	0.234007
Fine grain groats	2.467 (0.834)	2.929 (0.616)	0.171789
Ready-made breakfast products	2.200 (1.320)	2.714 (0.994)	0.251712
Oils	3.733 (0.961)	3.500 (1.019)	0.504539
Butter	3.933 (1.580)	3.929 (1.269)	0.880478
Margarine	1.333 (0.900)	1.571 (0.756)	0.270299
Cream	2.600 (1.298)	2.214 (0.975)	0.400494
Other animal fats	1.333 (0.724)	1.357 (0.633)	0.779995
Mayonnaise and dressings	2.333 (0.900)	2.643 (0.929)	0.376588
All fruit	4.133 (1.125)	4.286 (0.914)	0.714830
Stone fruit	2.733 (0.704)	2.714 (0.914)	0.714830
Kiwi and citrus	3.000 (1.363)	3.571 (0.938)	0.234007
Tropical fruit	2.067 (1.100)	2.286 (0.726)	0.376588
Berries	2.333 (0.900)	2.786 (0.975)	0.353554
Bananas	3.067 (1.033)	3.286 (1.069)	0.747192
Apples and pears	3.200 (1.474)	3.714 (1.139)	0.310143
Avocado	1.467 (0.743)	2.071 (0.917)	0.076919
Olives	1.733 (1.033)	2.214 (0.893)	0.158309
Dried fruit	2.267 (1.335)	2.357 (1.151)	0.779995
Sweet fruit preserves	2.400 (0.986)	2.429 (0.938)	0.982865
All vegetables	4.200 (1.373)	4.857 (1.027)	0.201205
Cruciferous vegetables	2.267 (0.961)	3.000 (0.679)	0.051090
Yellow-orange vegetables	2.533 (0.915)	3.929 (0.829)	0.000276
Green leafy vegetables	2.933 (1.163)	3.786 (0.802)	0.093216
Tomatoes	3.400 (1.352)	4.357 (0.745)	0.045875
Vegetables such as cucumber	2.600 (1.056)	3.643 (0.745)	0.009115
Root vegetables	2.933 (1.100)	3.786 (0.975)	0.051090
Potatoes	3.667 (0.900)	3.500 (0.650)	0.400494
Fresh legume seeds	2.200 (0.941)	2.571 (0.938)	0.331405
Dry legume seeds	1.867 (0.834)	2.286 (0.994)	0.310143
Nuts	2.800 (1.320)	3.214 (1.051)	0.251712
Seeds	1.800 (0.941)	2.286 (0.914)	0.158309
Sausages	2.467 (1.187)	3.000 (1.038)	0.270299
Cured meats	2.867 (1.356)	3.500 (1.019)	0.217176
Sausage products and organ meat	1.733 (0.961)	1.857 (0.949)	0.747192
Red meat	2.667 (1.047)	2.500 (1.092)	0.682954
Poultry and rabbit meat	3.133 (0.915)	3.429 (0.938)	0.310143
Venison	1.133 (0.352)	1.071 (0.267)	0.779995
Lean fish	2.267 (1.100)	2.143 (0.663)	0.813179
Oily fish	2.200 (0.941)	2.286 (0.825)	0.846694
Fruit juices and nectars	3.200 (1.207)	3.143 (0.535)	0.779995
Vegetable and vegetable and fruit juices	1.600 (0.828)	2.143 (1.099)	0.201205
Energy drinks	1.933 (1.223)	1.857 (1.099)	0.880478
Sweetened beverages	2.667 (1.496)	2.429 (1.453)	0.651619
Beer	1.800 (1.082)	2.000 (1.038)	0.590745
Wine and drinks	1.667 (0.900)	2.500 (0.855)	0.017844
Vodka and hard alcohol	1.333 (0.488)	1.929 (0.829)	0.062953

^1^ based on the Student’s t test; ^2^ based on the Mann–Whitney U Test; SD—standard deviation; S—the study group; C—the control group.

**Table 3:** Comparison of the average daily consumption of various nutrients in the study and control groups.

	S (*n* = 10)		C (*n* = 11)	*p*-Value ^1^	*p*-Value ^2^	*p*-Value ^3^
	A					
Energy (kcal)	1848.34 (885.49)	1629.68 (829.14)	2216.08 (596.84)	0.129617	0.274265	0.076456
Protein (g)	71.93 (36.52)	62.23 (24.83)	93.34 (20.53)	0.334764	0.109819	**0.005365**
Fats (g)	58.91 (37.18)	55.06 (26.43)	79.13 (25.50)	0.597186	0.159118	**0.04707**
Carbohydrates (g)	268.18 (122.92)	229.68 (142.76)	291.98 (93.97)	**0.037566**	0.621827	0.247834
SFA (g)	24.1 (16.64)	23.04 (12.28)	29.28 (12.37)	0.708677	0.425038	0.261382
MUFA (g)	21.12 (12.93)	20.19 (9.56)	31.28 (10.95)	0.748712	0.066247	**0.0236**
PUFA (g)	8.83 (5.09)	7.29 (3.31)	12.13 (4.21)	0.275192	0.120986	**0.009078**
n – 3 (g)	1.68 (0.84)	1.62 (1.05)	2.49 (1.2)	0.802552	0.092528	0.095492
Fiber (g)	21.02 (9.43)	16.58 (6.92)	23.79 (4.71)	0.06617	0.397802	**0.01101**
Cholesterol (g)	263.43 (183.82)	244.95 (132.66)	346.6 (156.96)	0.649039	0.277337	0.12742
Sugar (g)	105.13 (86.78)	89.79 (105.37)	106.58 (74.84)	0.297065	0.967556	0.67605
Tryptophan (mg)	884.78 (475.61)	776.2 (306.24)	1185.53 (297.63)	0.430469	0.09532	**0.005833**
B9 (µg)	320.11 (182.43)	255.92 (126.25)	382.97 (126.77)	0.181217	0.36685	**0.033071**
B12 (µg)	4.63 (4.27)	2.88 (1.8)	4.3 (1.27)	0.1372	0.807196	**0.049312**
C (mg)	139.66 (80.08)	110.19 (69.04)	154.02 (87.88)	0.21693	0.701004	0.222366
A (µg)	1846.53 (1931.33)	904.84 (329.47)	1416.43 (504.93)	0.137721	0.483948	**0.013656**
D (µg)	2.01 (1.61)	1.9 (1.28)	2.83 (2.7)	0.764501	0.41533	0.332884
E (mg)	8.31 (3.95)	6.9 (3.21)	12.33 (2.56)	0.204891	**0.011688**	**0.000378**
Selenium (µg)	9.98 (12.9)	4.39 (4.49)	24.56 (43.11)	0.225044	0.317598	0.158082
Zinc (mg)	9.33 (4.58)	7.98 (3.81)	11.23 (1.81)	0.240153	0.21886	**0.020072**
Magnesium (mg)	292.96 (139.06)	253.84 (120.38)	362.29 (54.33)	0.248926	0.141767	**0.014033**
Iron (mg)	11.26 (5.91)	8.35 (4.24)	12.68 (2.35)	**0.029401**	0.471301	**0.008538**

Student’s *t* test; ^1^ *p*-value for the difference in consumption between the study group before and after ketamine treatment; ^2^ *p*-value for the difference in consumption between the pre-ketamine and control groups; ^3^ *p*-value for the difference in consumption between the study group after ketamine treatment and the control group; S—study group; C—control group; B—study group before ketamine treatment; A—study group after ketamine treatment.

**Table 4:** Coverage of the daily requirements for individual nutrients by group.

Coverage of the Daily Requirement	Before (*n* = 10)	After (*n*=10)	C (n = 11)
n − 3	20%	30%	54%
Fiber	40%	20%	36%
Tryptophan	90%	70%	100%
Selenium	0%	0%	9%
Zinc	70%	40%	100%
Magnesium	40%	30%	73%
Iron	40%	40%	45%
B12	70%	60%	91%
B9	40%	30%	36%
Vitamin C	70%	60%	73%
Vitamin A	60%	50%	100%
Vitamin D	0%	0%	0%
Vitamin E	60%	30%	91%

Before–the study group before ketamine treatment; After–the study group after ketamine treatment; C–the control group.

## Data Availability

The data that support the findings of this study are available from the corresponding author upon reasonable request.
